# Spatiotemporal properties of whisker-evoked tactile responses in the mouse secondary somatosensory cortex

**DOI:** 10.1038/s41598-020-57684-6

**Published:** 2020-01-21

**Authors:** Sophie Hubatz, Guillaume Hucher, Daniel E. Shulz, Isabelle Férézou

**Affiliations:** 1 Department of Integrative and Computational Neuroscience (ICN), Université Paris-Saclay, CNRS, Institut des Neurosciences Paris Saclay, Gif-sur-Yvette, 91190 France; 2 Institut de biologie de l’École Normale Supérieure (IBENS), École Normale Supérieure, CNRS, INSERM, PSL Research University, Paris, 75005 France

**Keywords:** Sensory processing, Cortex, Whisker system

## Abstract

The representation of rodents’ mystacial vibrissae within the primary somatosensory (S1) cortex has become a major model for studying the cortical processing of tactile sensory information. However, upon vibrissal stimulation, tactile information first reaches S1 but also, almost simultaneously, the secondary somatosensory cortex (S2). To further understand the role of S2 in the processing of whisker inputs, it is essential to characterize the spatio-temporal properties of whisker-evoked response dynamics in this area. Here we describe the topography of the whiskers representation in the mouse S2 with voltage sensitive dye imaging. Analysis of the spatial properties of the early S2 responses induced by stimulating individually 22 to 24 whiskers revealed that they are spatially ordered in a mirror symmetric map with respect to S1 responses. Evoked signals in S2 and S1 are of similar amplitude and closely correlated at the single trial level. They confirm a short delay (~3 ms) between S1 and S2 early activation. In both S1 and S2 caudo-dorsal whiskers induce stronger responses than rostro-ventral ones. Finally, analysis of early C2-evoked responses indicates a faster activation of neighboring whisker representations in S2 relative to S1, probably due to the reduced size of the whisker map in S2.

## Introduction

Since the first description of its remarkable cellular organization by Woolsey and Van der Loos^1^, the rodent primary somatosensory (S1) cortex has become a major model for studying the cortical treatment of tactile sensory information^2–4^. In its layer 4, neurons form clusters, called barrels, that share the same topology as the whiskers on the snout of the animal. Each neuronal column associated with a barrel receives primarily the input coming from its corresponding whisker. Upon tactile stimulation of a given whisker, information is indeed rapidly transmitted to its corresponding barrel in S1, but also, within the next couple of milliseconds, to the secondary somatosensory cortex S2^5–8^. The rapid activation of S2 neurons following whisker stimulation is likely to rely on the direct projections it receives from both the ventral posteromedial nucleus^9–11^ and the posteromedial complex of the thalamus^12–14^. Hence, although S2 is usually considered as a higher order area compared to S1, it seems that cortical processing of tactile information follows a parallel, rather than hierarchical, scheme in rodents. The S2 cortex is nonetheless densely interconnected with S1, in a reciprocal and topographic manner^8,15–21^.

Electrophysiological recordings made in S2 in rats and mice show generally larger receptive fields than in S1, with an absence of single-whisker receptive fields^5,22–24^. Analysis of responses to complex multi-whisker stimuli in anesthetized rats has recently revealed that S2 neurons are likely to integrate sensory information over larger time and spatial scales in comparison to S1 neurons^23^. Studies on behaving mice have suggested that a representation of the tactile scene could emerge through coordinated activity between S1 and S2^25–28^. However, knowledge about the involvement of S2 in the processing of tactile sensory stimuli originating from the whiskers remains limited.

The topography of the whiskers representation in S2 has been functionally explored in rats using surface electrodes^6,29^. In mice however, no quantitative exploration of the whisker representation is available (but see^22^ for a non-quantitative study).

In order to provide a thorough description of the topography of the whiskers representation in the mouse S2, and a quantitative comparison of the dynamics of single whisker-evoked responses between S1 and S2, we used simultaneous voltage sensitive dye (VSD) imaging of both cortical areas in anesthetized mice. This technique indeed resolves the dynamics of cortical activity with a spatial resolution of a few tens of micrometers at the millisecond timescale^30–33^.

## Results

### Resolving spatio-temporal dynamics of whisker-evoked neuronal activity over both S1 and S2 by means of VSD imaging

**Figure 1:**
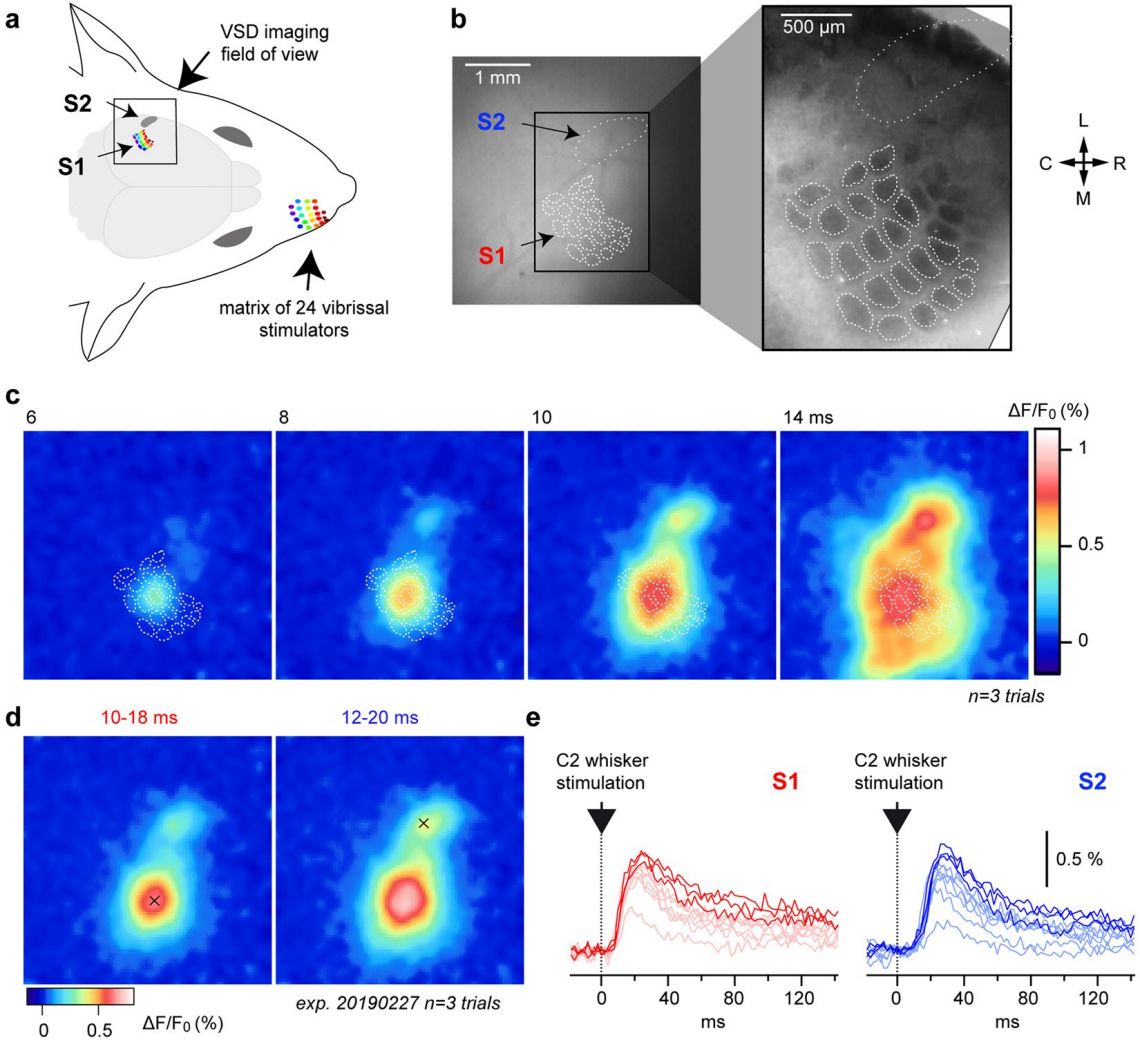
Voltage sensitive dye imaging of whisker-evoked spatio-temporal dynamics in both S1 and S2. (**a**) Experimental setup. The left S1 and S2 cortical areas are imaged at 500 images per second using a high-speed imaging system while the whiskers on the right side of the snout are stimulated with a matrix of 24 vibrissal piezoelectric actuators. (**b**) The surface of the cortex illuminated at 630 nm over the imaging field of view reveals the fluorescence of the dye RH1691 (left). The white dotted line indicates the S2 area location as defined from functional responses (c.f. Fig. 2). The white dashed lines outline the contour of the S1 layer 4 barrels defined from a post-hoc histological cytochrome oxidase staining (right). The barrel map was aligned with VSD images using the surface blood vessels as anatomical landmarks. L: lateral, M: medial, R: rostral, C: caudal. (**c**) Snapshots of the cortical activity imaged in an anesthetized mouse (isoflurane) at different timings after the start of a brief stimulation of the right C2 whisker (data averaged from 3 highest response amplitude selected trials out of 10 and Gaussian filtered [7 × 7 pixels]). The histologically reconstructed barrel field is shown as white  dashed outlines. (**d**) The center of the functional representations of the C2 whisker in S1 and S2 for this mouse was determined from the same dataset by averaging the images over a time window of 10 to 18 ms for S1 (left), and 12 to 20 ms for S2 (right), and pinpointing local maxima (shown as black crosses). (**e**) Fluorescence profiles (ΔF/F_0_) measured in response to 10 C2 stimulations in S1 (red, left) and S2 (blue, right) from regions of interest (ROIs) of ~115 × 115 μm, centered on the C2 representation in S1 and S2, respectively (black crosses in **d**). Bright colors indicate the 3 selected trials for the averaged images illustrated in **c,d**, the other trials are illustrated in faint colors.

A large region of the mouse cortex covering both S1 and S2 was exposed and stained with the dye RH1691. This VSD allows reporting at the millisecond timescale ensemble subthreshold membrane potential variations from supragranular layers^30,34^. We used this experimental approach to study the spatio-temporal dynamics of responses evoked by single-whisker stimuli delivered by means of a matrix of 24 piezoelectric whisker stimulators (Fig. 1a). At the end of the imaging sessions, tangential brain slices were stained for cytochrome oxidase in order to reconstruct the S1 barrel map, which can be then overlaid to the functional images by using surface blood vessels as anatomical landmarks (Fig. 1b)^35^. Note that over the nine animals included in this study, we did not observe any noticeable cytochrome oxidase reaction over the S2 area. The single brief caudal deflection of the central C2 whisker induced a rapid excitation of S1 which was initially centered on the C2-related barrel and then rapidly spreads over the entire S1 barrel cortex, as previously reported^30^. This initial depolarization was followed, in the next few milliseconds, by a second localized signal emerging rostro-laterally relative to the first response, within S2. Due to the fast propagation of the second depolarization, the signals originating from S1 and S2 rapidly merge at the border between the two areas. The Fig. 1c illustrates the dynamics of the C2 whisker-evoked signals from an average of three high amplitude response trials in a representative experiment. Local maxima from early localized tactile responses were used to define the center of the C2 whisker’s representation in both S1 and S2 (Fig. 1d). They could then be used as the centers of regions of interest (ROIs) used to quantify the temporal dynamics of the fluorescent signals from S1 and S2 (Fig. 1e).

### Somatotopic organization of tactile responses in S2

**Figure 2:**
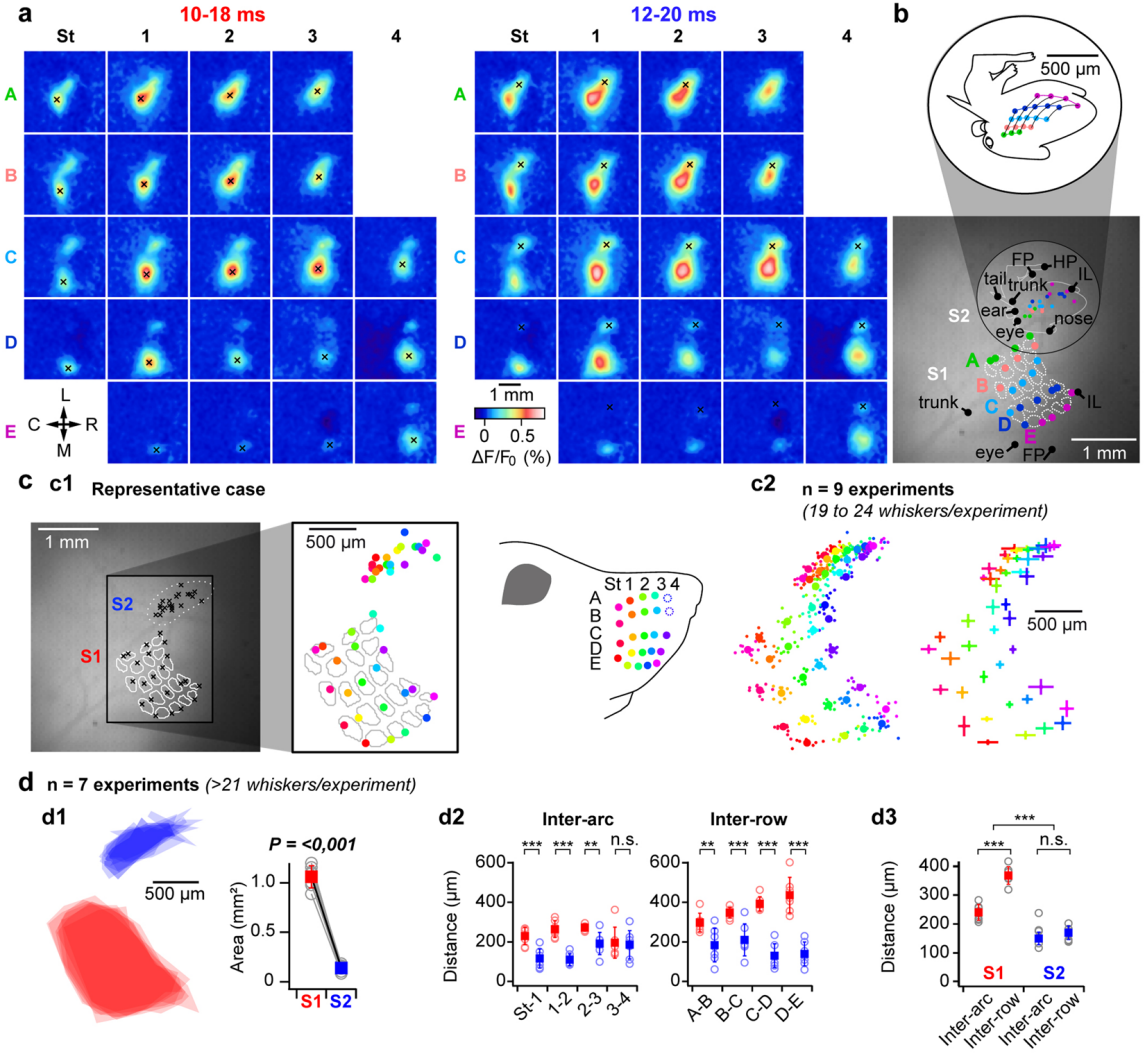
Functional mapping of whiskers representation in S1 and S2. (**a**) Functional mapping of the representation of 22 whiskers in S1 and S2 was performed by analyzing VSD images of early cortical responses to single whisker deflections (as illustrated for C2 of the same animal in Fig. 1: n = 3 highest response amplitude trials over 10), local maxima indicated by black crosses are used as proxy for the center of functional representations in S1 and S2 (note that the S2 representation of A3 is not shown as it could not be distinguished from its S1 representation). L: lateral, M: medial, R: rostral, C: caudal. (**b**) For the same animal, responses to tactile stimuli delivered to different parts of the body (inferior lip [IL], eye, ear, trunk, tail, forepaw [FP], hind paw [HP], were also recorded. Local maxima extracted from early signals are shown together with the whisker representations in S1 and S2, over the reference fluorescent image (bottom, white dashed lines outline the contours of the S1 layer 4 barrels) allowing the reconstruction of a S2 “*mousunculus*” (white solid line). Top: Sketch of the S2 *mousunculus* (**c**) The functional mapping of whiskers representation reveals a clear somatotopy within S2. c1: same experiment than in **a**, the center of the functional representations of the 22 whiskers in S1 and S2 are shown on the reference fluorescence image (left). To better visualize the functional mapping, the identity of the whiskers has been color-coded (right, note the absence of A4 and B4 for this animal). c2: Data presented for 9 similar experiments (n = 19 to 24 whiskers per experiment) and realigned relative to the S1 and S2 centroids, respectively (centroids calculated over the 18 whiskers that are common to all the experiments). Left: individual maps (small dots) together with averaged map (filled circles). Right: Averaged map +/− standard deviations. (**d**) Comparison of S2 versus S1 functional maps (n = 7 mice with ≥21 whiskers). d1: Superimposition of S1 (red) and S2 (blue) areas determined by joining the locations corresponding to the most external whiskers (excluding A3, A4, B4) with the corresponding quantification (right, Paired t-test comparison). d2: Quantification of individual inter-row and inter-arc distances. d3: Averaged inter-row and inter-arc comparison. n.s.: non-significant **p < 0.01, ***p < 0.001 (two way repeated measures analysis of variance, Holm-Sidak multiple comparison).

Using such experimental approach, we could, by deflecting single whiskers within pseudo-randomized sequences (n = 10 trials per whisker, 3 trials with the highest response amplitude selected), reveal the spatial organization of whiskers representation within both S1 and S2 (Fig. 2a, same animal as in Fig. 1). One can note that this animal was missing A4 and B4 whiskers, which are frequently absent in this mouse strain. In addition, the S2 representation of the whisker A3 could not be established since it was too close to its S1 representation, reaching the spatial limitation of our method. For this animal, we further recorded responses to tactile stimuli applied to different parts of the body which allowed drawing its “*mousunculus*” (Fig. 2b), whose global position and orientation is in agreement with previously reported data from rats^6^ and mice^22^. Color-coding the identity of the stimulated whiskers reveals a clear topographic arrangement of whiskers representation within S2 (Fig. 2c, n = 9 mice, note that the number of considered whiskers varied from 19 to 24 per animal, S1 and S2 whisker maps are therefore realigned, respectively according to the S1 and S2 centroids calculated over the 18 whiskers that were common to all experiments). This secondary whisker map is organized in a mirror symmetric manner with regard to the S1 barrel-map, lying rostro-laterally to the primary barrel cortex (the central C2 whisker’s representation within S2 lies 350 +/− 54 µm rostrally, and 1254 +/− 143 µm laterally, from its representation within S1, n = 9).

Overall, the whisker map covers a much smaller extend of the cortical surface in S2 than in S1 (12.84 +/− 2.19% relative to S1 barrel map, n = 7), both inter-arc and inter-row distances being reduced in S2 relative to S1 (Fig. 2d). Although the representations of individual rows of whiskers are more separated than those of individual arcs within S1, reflecting the spatial configuration of the whiskers implantation at the periphery, this difference is not significant in S2 where the spatial organization of the map seems distorted with regard to the geometry of the whisker pad.

### Comparison of whisker-evoked responses in S2 and S1

**Figure 3:**
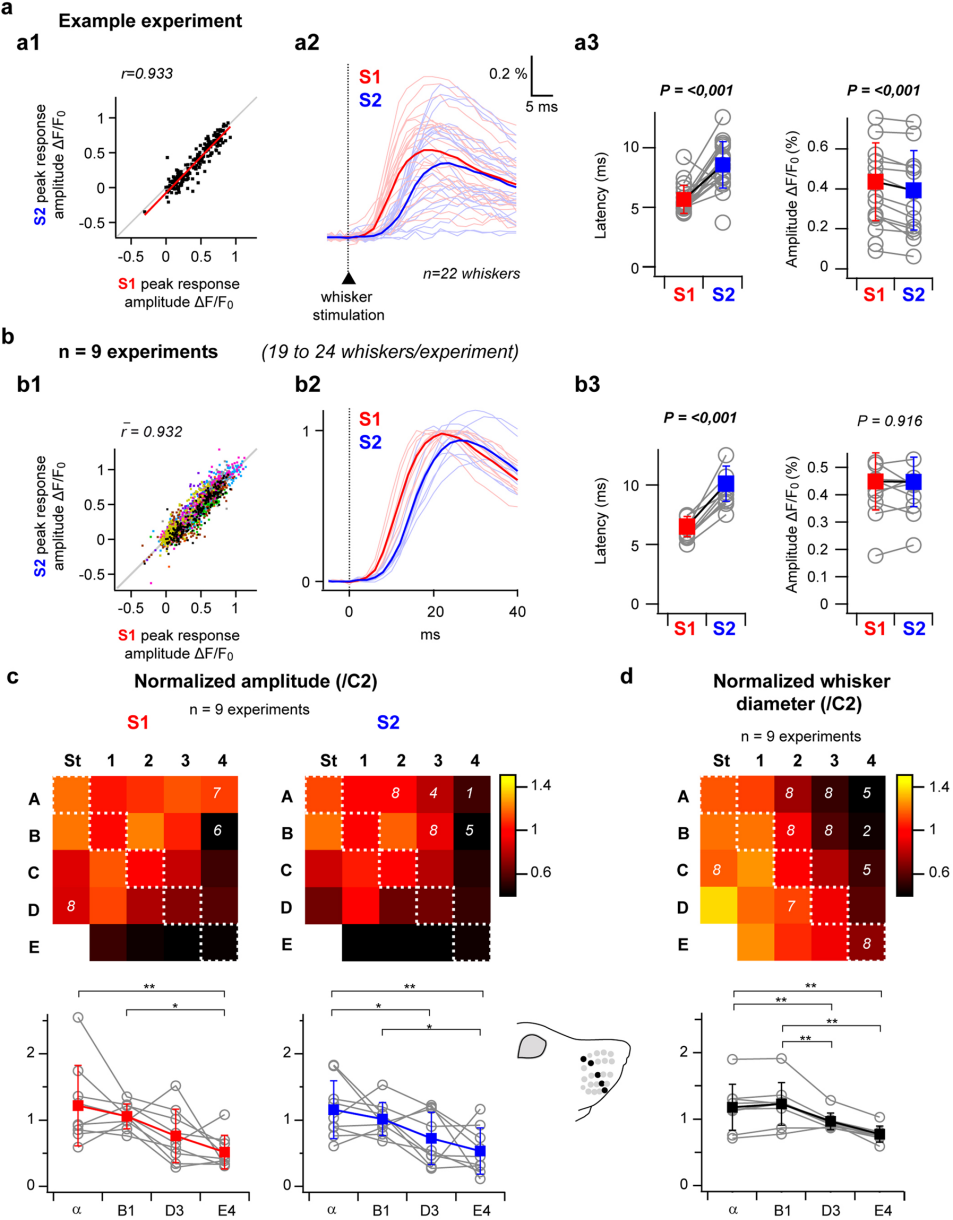
Comparison of whisker-evoked responses in S2 versus S1. (**a**) Whisker-evoked responses in S2 versus S1 for a single example experiment (same as in Figs. 1 and 2). a1: Single trial response amplitudes in S2 plotted as a function of responses amplitudes measured in S1 (n = 22 whiskers × 10 trials) demonstrate a close to linear relationship. a2: Averaged fluorescence profiles showing responses to each individual whisker (faint colors), together with all-whiskers averages (bright colors). a3: S2 versus S1 comparison of responses latencies (quantified by extrapolation of a linear 20–80% fit of the rising phase of the signal) and amplitudes, grey open circles show averaged values for individual whiskers over n = 10 trials, filled red and blue squares show all-whiskers averages +/− standard deviations in S1 and S2, respectively. (**b**) Same analyses applied to the population (n = 9 experiments). In b1, each color represents an individual experiment; b2, all-whiskers averages are shown for each experiment after normalization by the peak of S1 response. In b3, only all-whisker averages are shown as grey open circles for each experiment, filled squares show averages across the 9 experiments (+/− standard deviation). (**c**) Top: S1 and S2 whisker maps illustrating the amplitudes of averaged whisker-evoked responses normalized by the C2-evoked response amplitude (n = 9 experiments, n = 10 trials per whisker, white numbers on the maps indicate lower n values for some whiskers due to either absent whisker or impossible determination of the corresponding cortical representation location). These maps reveal both in S1 and S2 a gradient of response intensities throughout the barrel map. Bottom: comparison of the amplitudes along a diagonal of the whisker map reveals significant gradually decreasing responses from α to E4. (**d**) Top: whisker diameters measured at the skin-insertion level and normalized by the C2 whisker diameter (n = 9 mice, white numbers indicate lower n values for some whiskers that were either absent or lost during the experimental procedure). Although they also gradually decrease from α to E4, the overall gradient of whiskers diameter seems to present an orthogonal axis relative to the response amplitude maps. *p < 0.05, **p < 0.01 (Friedman repeated measures analysis of variance on ranks or one way repeated measures analysis of variance, Tukey Test multiple comparison).

Quantification of the fluorescent signals from ROIs defined by using the above-described mapping method was used to compare responses to individual whisker stimulations in S2 versus S1 (Fig. 3). Within the S1 barrel cortex, such responses have been reported to be highly variable from trial to trial due to interactions with spontaneous depolarizations^36,37^. Moreover, it has been recently proposed that this variability could take part in a coding scheme where the strength of a tactile stimulus would be encoded by the likelihood of the cortical response rather than its magnitude, in a framework referred to as the “probability of activation hypothesis”^38^. We therefore quantified the sensory-evoked response amplitudes in S1 and S2 for each single trial. Our results reveal a close to linear relationship between S1 and S2 responses that co-varied trial by trial, with large signals in S2 accompanying large signals in S1 (Fig. 3a1,b1**)**. The variability of responses in S1 and S2 therefore seems to be governed by the same mechanisms.

For the same experiment as in Figs. 1 and 2, the averaged profiles of fluorescence evoked by individual whisker stimuli (n = 10 trials per whisker, Fig. 3a2) reveal responses of higher latencies in S2 than in S1 (Fig. 3a3). At the population level (n = 9 mice), responses in S2 presented an overall small but significant delay in latency (+3.3 +/− 1.2 ms, Fig. 3b2,b3), consistently with recently reported electrophysiological data^8^. Although the amplitude of evoked responses was smaller in S2 than in S1 for the illustrated case (Fig. 3a3), this was not true for all the experiments and consequently, we did not observe a significant difference in response amplitude between the two areas over the population (Fig. 3b2,b3).

In both S1 and S2, the intensity of cortical activation differs according to the identity of the stimulated whisker. Response amplitude maps (Fig. 3c) reveal a gradient following the E4 to alpha axis, with weaker activations recorded in response to the stimulation of rostro-ventral whiskers and stronger responses evoked by the stimulation of caudo-dorsal whiskers. To assess if this gradient could rely on an unevenness in the distribution of angular tuning throughout the cortical whisker representations, four additional experiments in which S1 cortical responses to whisker stimuli consisting of individual deflections of 22 to 24 whiskers in the 4 cardinal directions were performed (Supplementary Fig. S1). These experiments failed to reveal a clear spatial distribution of the direction preference for single whisker deflections, and response amplitude maps obtained by averaging together the responses to stimuli delivered in the 4 directions revealed a similar E4 to alpha gradient as the one illustrated in Fig. 3c. It is therefore unlikely that a spatial bias in directional tuning is generating the observed gradient in whisker-evoked responses.

A variation of the diameter of the whiskers could affect the cortical responses since a thicker whisker might exert more torque and induce stronger activation of primary sensory neurons.Throughout the large caudal whiskers under study here, and often referred to as macrovibrissae, the diameter is not an uniform feature, caudal whiskers appearing thicker than rostral ones^1^. To assess a possible link between the observed gradient in whisker-evoked cortical responses and the way tactile inputs are generated at the periphery, we quantified for each experiment the whiskers diameter at their skin insertion point (Fig. 3d). Our quantification reveals a gradient in diameter in the A4 to delta axis, from thin dorso-rostral whiskers to thick ventro-caudal ones, which matches the gradient previously reported for of rat vibrissal follicle diameters^39^, as well as the gradient described in mice relative to the innervation density of whisker follicles^40^. Although there is a tendency for diameter augmentation along the alpha to E4 axis, the overall gradient is orthogonal to the one observed for whisker-evoked responses.

### Spatial propagation of signals evoked by the central C2 whisker in S1 and S2

**Figure 4:**
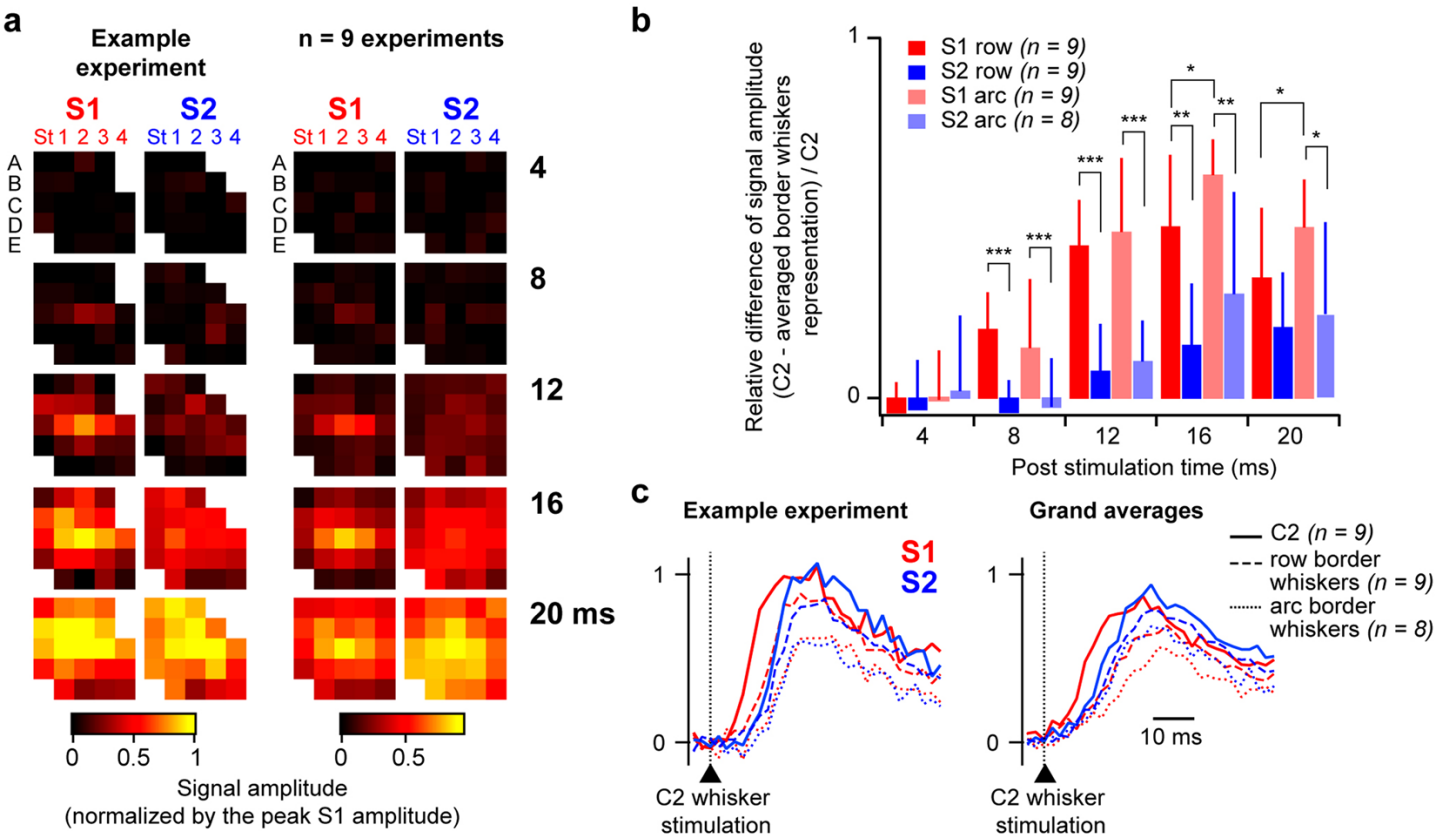
Spatial propagation of signals evoked by the central C2 whisker in S1 and S2. (**a**) Left: to characterize early propagation of single whisker-evoked signals to neighboring whiskers representations in S1 and S2, signals evoked by the central C2 whisker stimulation (averaged from n = 10 trials) were quantified from ROIs (single pixel size) centered on each whisker’s representation in both S1 and S2, as determined by the functional mapping illustrated in Fig. 2 for this experiment. After normalization by the peak response in S1, signal amplitudes at different timings relative to the whisker deflection were measured at each location (color-coded, and represented in standard matrixes. Right: same analysis applied to the population (n = 9 experiments,19 to 24 whiskers/experiment). (**b**) To compare the propagation of evoked activity along two cardinal orientations (row C versus orthogonal arc 2) we extracted the values corresponding to gamma, C2, C4, A2 and E2 from the analysis presented in **a**. The relative difference between the signal measured at the central C2 representation and the averaged signal from the borders of the whisker map along the row C (gamma and C4) or the arc 2 (A2 and E2), over the signal measured at C2, was used to quantify the propagation of whisker relative information in S1 versus S2. *p < 0.5, **p < 0.001, ***p < 0.001 (two way repeated measures analysis of variance, Holm-Sidak multiple comparison). (**c**) Left: for the same experiment as in (**a)**, signals recorded in response to the central C2 whisker stimulation in S1 (red) and S2 (blue) were either quantified from the ROI corresponding to the C2 whisker representation (solid lines, n = 10 trials), or averaged from distant ROIs along the row C (gamma and C4, n = 10 trials each, dashed lines) and the arc 2 (A2 and E2, n = 10 trials each, dotted lines). Right: grand averages over the population.

To study how the information arising from a single whisker at the periphery propagates to neighboring whiskers representation within S1 and S2, we chose to focus our attention on cortical responses evoked by the stimulation of the central C2 whisker. Measuring signal amplitude from ROIs centered on each whisker’s representation in both S1 and S2, as determined by the functional mapping described above, revealed distinct dynamics in S2 compared to S1(Fig. 4). As previously reported^30^, individual C2 whisker stimulation results, in S1, in an activation first restricted to the corresponding barrel-related column, which then rapidly spreads to the surrounding columns. In contrast, the activation in S2 seems much less localized even at the early onset of the response (Fig. 4a). Within S1, the propagation of tactile sensory information has been described to be faster along the row axis relative to the orthogonal arc axis^41–43^. In order to eventually quantify such directional preference in S1 and S2, we computed the relative difference between the signal measured at the central C2 representation and the averaged signal from the borders of the whisker map along the row C (gamma and C4) or the arc 2 (A2 and E2), over the signal measured at C2 (Fig. 4b). Such ratio would tend to 0 in the hypothetic case where the C2 whisker stimulation would similarly activate its related cortical domain and the borders of the map, or to 1 in the extreme case where it would result in activating only its corresponding cortical representation area with no impact on the cortical domains corresponding to remotely located whiskers. Our results confirm a preferential propagation of the information in the row axis within S1, with ratios significantly smaller in the row axis than in the arc axis at 16 and 20 ms following the whisker deflection. Smaller values were overall measured in S2, revealing an overall more diffused activation of the map relative to S1. Finally, like in S1, ratios tend to be smaller in the row axis than in the arc axis, although this difference is not statistically significant in S2. To ease the visualization of these temporal dynamics, we plotted the fluorescence profiles quantified from the C2 ROIs in S1 and S2, together with the averaged signal from ROIs corresponding to border whiskers along the row C (gamma and C4) or the arc 2 (A2 and E2, Fig. 4c). These traces indeed show that the C2 whisker stimulation first evokes a signal in S1, which then propagates faster along the row C than the arc 2 axis. While the S2 response starts in C2 with a little delay, it then invades the whole whisker map very rapidly, along both axes. Following the deflection of a single central whisker, subthreshold activation of the cortical representation of the whiskers located at the extreme borders of the whisker array is consequently not faster in S1 than in S2. This is most likely due mainly to the reduced size of the whisker map in S2, since quantification of the propagation velocity in absolute cortical distance did not reveal any difference between S1 and S2 (Supplementary Fig. S2).

## Discussion

By taking advantage of the high spatiotemporal resolution of VSD imaging^31–33^ on the one hand, and of the ability to tightly control independent whisker deflections by using a dedicated matrix of 24 piezoelectric stimulators^44^ on the other hand, this study provides a systematic comparative description of the mouse whiskers’ representation in the secondary versus the primary somatosensory cortex.

In agreement with reported electrophysiological data^22^, the overall body map in the mouse S2 lies laterally from S1 with a mirrored topographic organization, global features that seem to be shared with other rodent species^6,45,46^. Stimulation of up to 24 individual whiskers in 9 mice revealed that although the cortical territory devoted to whiskers in S2 is about an order of magnitude smaller than in S1, it is characterized by a clear somatotopic organization with a discernable topographic arrangement of individual whiskers.

Because VSD imaging reports averaged subthreshold membrane potential variations originating mainly from supragranular layers, the signals evoked by a single whisker deflection rapidly spreads laterally both in S1 and S2. However, thanks to the high temporal resolution of the technique, by focusing on the early phase of the responses, we could resolve single whisker representations within both S1 and S2.

The mouse neocortex therefore counts three distinct ordered maps for processing tactile inputs originating from their macrovibrissae, one in S1, one in S2 and one in the primary motor cortex (M1)^47^, which are activated in that sequential order. The largest map located in S1 presents spatial properties that reflect the spatial arrangement of the tactile sensors at the periphery, with more distance between rows than between arcs. While this asymmetry is also present in M1, where the whisker sensory map is about half the size of that of S1^47^, it appears to be lost in S2, as we did not observe any significant difference between inter-row versus inter-arc distances in the whisker map, which is overall about ten times smaller than in S1.

Recordings of S2-projecting versus M1-projecting neurons in S1 in awake mice involved in operant conditioning tasks have raised the hypothesis that S2 and M1 could play distinct roles in the processing of tactile sensory information. Propagation of touch related signals towards M1 may be important for object localization while a stream of information involving S2 may be particularly involved in the identification of object features and sensorimotor transformations^25,26,48,49^. In line with this hypothesis, one could note that a broader whisker map in M1, which respect the spatial arrangement of whiskers at the periphery is likely to optimize object localization. On the other hand, the fastest activation of S2, which presents a compacted whisker map, might favor multiwhisker integration and efficient coding of object’s texture. Recent electrophysiological data from the rat S2 have indeed reported that multi-whisker integration tends to be more supra-linear in S2 than in S1^23^. Moreover, a specific implication of S2 in bilateral integration is suggested from its connections with contralateral S1 and S2^9^ and from experience dependent plasticity experiments^50^. It would be therefore of particular interest in the future to compare, by means of similar VSD imaging experiments, cortical dynamics evoked by both contra- and ipsi-lateral multiwhisker stimuli in S2 versus S1.

In response to individual whisker stimuli, we observed a tight correlation between S1 and S2 response amplitudes at the single trial level demonstrating that under anesthesia common mechanisms are shaping the responses in the two areas. However, through learning of an operant conditioning task, activity in S2 has been shown to be more associated with the perceptual outcome than in S1^25,28^. The relationship between S1 and S2 evoked-dynamics might therefore be more complex when tactile stimuli are delivered in the context of a goal directed task.

We observed a shared gradient of whisker-evoked response amplitudes throughout the whisker array in S1 and S2, with caudo-dorsal whiskers inducing stronger responses than rostro-ventral ones. Quantification of whisker diameters revealed an orthogonal gradient, from thick caudo-ventral to thin rostro-dorsal whiskers, consistently with reported measures of rat whisker follicle diameters^39^. The gradient in evoked response amplitudes is therefore not directly correlated to whisker diameter. Quantitative studies have been performed in the rat to assess how the cellular organization of a given vibrissa representation along the principal path of information flow, from the periphery to the S1 barrel cortex, differs according to its identity^39,51^. They reported dissimilar gradients in barreloid size^39^ and volume^51^ in the ventral posterior medial division of the thalamus increasing either from alpha to E4^39^ or from A4 to delta^51^ (similarly to cortical barrel-related column volume^51^, and whisker follicle diameter^39^).

These observations, at odds with our functional imaging data, suggest that the number of neurons collecting information from a given whisker is most probably not the only parameter conditioning the magnitude of cortical sensory responses. Although it is important to note that no precise similar stereological quantification covering the full whisker array has been performed on C57Bl6 mice.

Finally, by measuring how responses to the central C2 whisker activates distant whisker’s cortical representation in S1 and in S2, we observed a faster lateral propagation of sensory information within the whisker map in S2 than in S1. This is most probably linked to the compacted whisker map in S2, and might rely on specificities in thalamo-cortical and cortico-cortical connectivity of this area. It has been recently demonstrated that projections from the layer 4 of S2 towards S1 are somatotopically organized and carry stimulus specific information, which can modulate orientation tuning in a subset of S1 neurons^8^. In light of our results, one can hypothesize that S2 projections to the barrel cortex could also play an important role for multiwhisker information and extraction of more global features of the tactile scene. For now, it is difficult to disentangle the relative importance of these S2 to S1 projections versus the intrinsic horizontal transcolumnar connectivity of the S1 barrel cortex regarding multiwhisker integration. Preference for the row axis in the lateral propagation of sensory-evoked signals in S1 matches observed biases in the intrinsic connectivity (although described essentially in the rat barrel cortex^43,52^), although it does not rule out an impact of inputs originating from S2. Future work investigating the effect of specific pharmacological or optical neuronal inactivation of S2 neurons on the processing of tactile inputs in S1 and vice versa will be essential to further understand the role of S2.

Overall, our findings reveal with an unpreceded precision that the representation of macrovibrissae within the mouse S2 is topographic. Upon individual whisker stimulation S2 responds with a depolarization that is similar in amplitude than the one observed in S1 but slightly delayed in time. Propagation of sensory information towards distant whisker representations is faster in S2 than in S1, which is likely to favor multiwhisker integration. Given that connectivity data can bring arguments to both parallel^10,12^ and hierarchical^8^ views of the processing of tactile sensory inputs in the rodent whisker system, technological advances allowing gathering of data synchronously in the two areas during whisker dependent goal directed tasks^25,27,28^ will certainly open new perspectives to better depict the functional specificity of S2 in the future.

## Methods

### Animals and surgery

Experiments were performed in accordance with the French and European (2010/63/UE) legislations relative to the protection of animals used for experimental and other scientific purposes. Experimental procedures were approved by the French Ministry of Education and Research, after consultation with the ethical committee #59 (authorization number: APAFIS#3561-2016010716016314). VSD imaging was performed on 1 female and 8 male 6–21 week-old C57BL6J mice under isoflurane (induction 3–4%, maintenance 1–1.5%) anesthesia. Paw withdrawal, whisker movement and eye-blink reflexes were suppressed by the anesthesia. A heating blanket maintained the rectally measured body temperature at 37 °C. The respiration of the mice was monitored with a piezoelectric device and the brain state monitored by using two epidural electrodes above the barrel cortex and the frontal cortex ipsilateral to the stimulated whiskers. A metallic fixation post was implanted on the occipital bone with cyanoacrylate glue and dental cement. A ~5 × 5 mm craniotomy was made to expose S1 and S2. Extreme care was taken at all times not to damage the cortex, especially during the removal of the dura.

### VSD imaging

The voltage-sensitive dye RH1691 (Optical Imaging Ldt, Israel) was dissolved at 1 mg/ml in Ringer’s solution containing (in mM): 135 NaCl, 5 KCl, 5 HEPES, 1.8 CaCl_2_, 1 MgCl_2_. It was topically applied to the exposed cortex and allowed to diffuse into the cortex over 1 hour. After removal of the unbound dye, the cortex was covered with agarose (0.5–1% in Ringer’s) and a coverslip. Cortical imaging was performed through a tandem-lens fluorescence microscope (SciMedia Ldt, USA), equipped with a couple of Leica PlanApo objectives, a 100 W halogen lamp gated with an electronic shutter, a 630 nm excitation filter, a 650 nm dichroic mirror, and a long-pass 665 nm emission filter. We set the field of view to either 3.8 × 3.8 mm (n = 6 experiments), by mounting a 1.6x objective (cortex side) and a 0.63× (camera side) on the microscope, or to 2.5 × 2.5 mm (n = 3 experiments), by using a 5x objective on the cortex side, and a 5x objective on the camera side. Images were acquired with a high-speed MiCam Ultima camera (SciMedia Ldt., USA) at 500 Hz, with a spatial resolution of either 38 × 38 or 25 × 25 µm. The illumination of the cortical surface started 500 ms before each image acquisition to avoid acquiring signal in the steeper phase of the fluorescence bleaching. Recordings were then of 1 second duration, with 200 ms baseline and 800 ms post stimulation. Variations of the fluorescence signals were initially recorded as variations over the resting light intensity (first acquired frame).

### Whisker stimulation

Deflections of the right 22 to 24 posterior macrovibrissae of the mice were performed using a multi-whisker stimulator^44^ at 0.1 Hz with pseudo randomized sequences containing blank trials (each stimulation being repeated 10 times). Whiskers on the right side were cut to a length of 10 mm and inserted, while keeping their natural angle, in 27G stainless steel tubes attached to piezoelectric benders (Noliac, Denmark), leaving 2 mm between the tip of the tube and the whisker base. Each whisker deflection consisted of a caudal 95 µm-displacement (measured at the tip of the tube), a 2 ms rising time, a 2 ms plateau and a 2 ms fall. The deflection amplitude of each actuator was calibrated using a laser telemeter (Micro-Epsilon, France) and specific filters were applied to the voltage commands to prevent mechanical ringing of the stimulators. The resulting initial deflection velocity was of 1270°/s.

### Histology

At the end of the imaging sessions, mice were perfused intracardially with saline followed by paraformaldehyde (4% in 0.1 M phosphate buffer). After an overnight post-fixation in paraformaldehyde, the brains were cut in 100 µm-thick tangential sections and stained for cytochrome oxidase revealing the layer 4 barrel map. Microphotographs of the tangential sections were registered and the barrel maps reconstructed using a method implemented in Matlab (MatWorks, USA), essentially as previously described^35^. Briefly, the registration was achieved by rigid transformations computed from automatically detected blood vessel cross-sections, using a robust variant of the classical iterative closest point method. The reconstructed barrel map was then generated by computing a nonlinear merging of the gradients from the registered images. Finally, the functional VSD data were aligned with the reconstructed barrel maps by using the superficial blood vessels as anatomical landmarks.

### VSD imaging analysis

Acquisition and data preprocessing were done using in-house software (Elphy, G. Sadoc, UNIC-CNRS). Further analyses were made using custom written routines in IgorPro (WaveMetrics, USA). For each experiment, all the unstimulated blank trials were averaged together and pixel by pixel double exponential fit of the averaged blank trials were subtracted from each trial to correct for bleaching related artifact.

Variations of the fluorescence signal are expressed as ΔF/F_0_, the averaged signal over three frames just preceding the stimulus being used as a reference (F_0_).

### Quantification of whiskers diameter

After intracardiac perfusion and extraction of the brain for histological procedures (see above), individual whiskers were gently pulled-out from the skin and embedded on a slide for microscopic observation. Their diameter was quantified under a 20x or 40x objective, just next to their follicle insertion.

### Statistical tests

Statistics were performed with the SigmaStat software (Systat, USA). Single factor comparisons were made by using one-way analysis of variance for repeated measures followed by the Holm-Sidak method for multiple comparisons or, if the normality test failed, by Friedman repeated measures analysis of variance on ranks followed by a Tukey Test for multiple comparisons. Two factors comparisons were made by means of two-way repeated measures analysis of variance followed by Holm-Sidak multiple comparison.

## Supplementary information

Supplementary Information.

## Data Availability

Data are available upon reasonable request.
