# Supplementary material for: Spatiotemporal properties of whisker-evoked tactile responses in the mouse secondary somatosensory cortex
Source: Sci Rep. 2020 Jan 21;10:763. doi: 10.1038/s41598-020-57684-6 (PMC6972923; doi:10.1038/s41598-020-57684-6)
Supplement: Supplementary file 1 — Supplementary Information. [file 41598_2020_57684_MOESM1_ESM.pdf]

Supplementary information

Spatiotemporal properties of whisker-evoked tactile responses in the mouse secondary somatosensory cortex

Sophie Hubatz, Guillaume Hucher, Daniel E Shulz, Isabelle Férézou

Supplementary Figure S1. Angular tuning for individual whisker deflections is not spatially organized at the supra-barrel scale throughout S1.

Supplementary Figure S2. Spatial propagation of the C2 whisker-evoked signals is similar in S1 and S2 when quantified in absolute distance over the cortical surface.

Figure S1

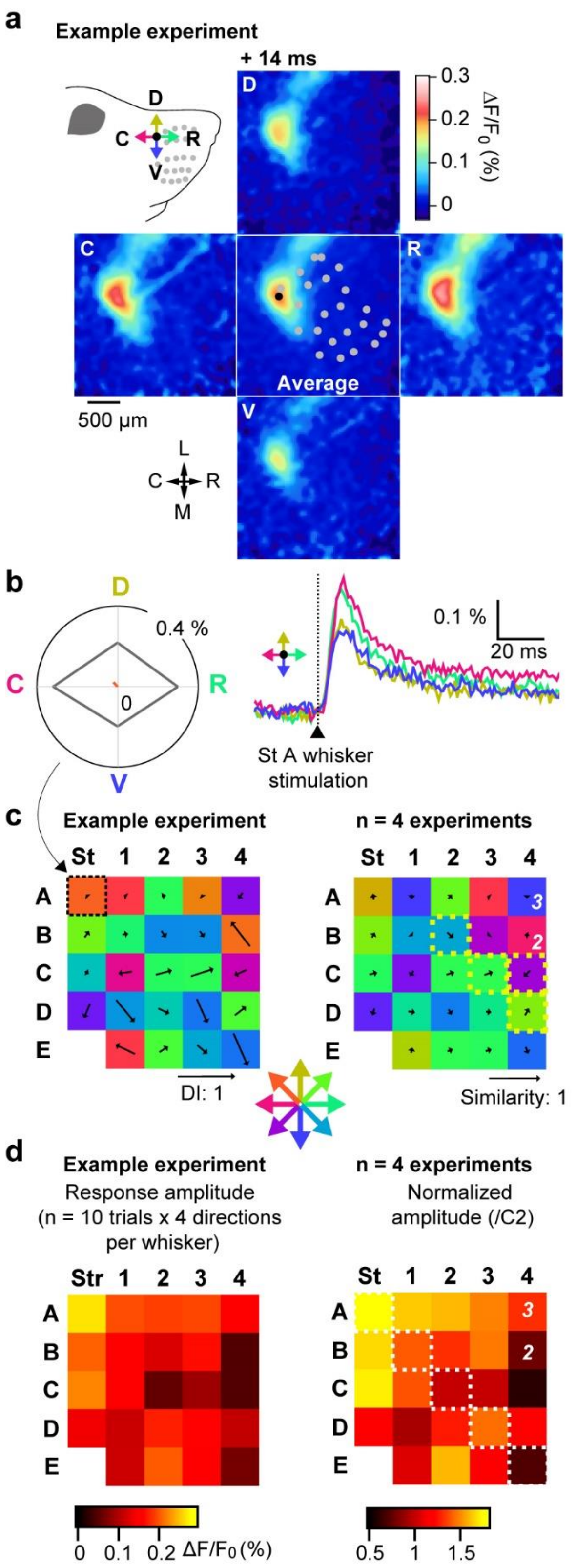

Figure S1. Angular tuning for individual whisker deflections is not spatially organized at the supra-barrel scale throughout S1.

The largest 22 to 24 whiskers of the right side of the snout were deflected individually, in the 4 cardinal directions, at 0.1 Hz with pseudo randomized sequences containing blank trials (each stimulation being repeated 10 times) in 6–12 week-old C57Bl6 mice (n = 4) under urethane anesthesia (1.7 mg/g). Evoked cortical activity was imaged at 500 Hz (25 µm/pxl) with the voltage sensitive dye RH1691, over a field of view of 2.5 x 2.5 mm covering S1.

(**a**) Cortical activity imaged at 14 ms following a single α-whisker (St A) deflection in the dorsal (D, up), ventral (V, bottom), rostral (R, right) or caudal (C, left) direction (averages of n = 10 trials). The central image is a grand-average over the 4 conditions. The black dot shows the localization of the representation of St A obtained from these data, and the grey dots show the mapping of the other 23 whiskers for the same animal.

(**b**) On the right are shown profiles of fluorescence computed from the black region of interest shown in **a**. Each profile shows the response to one of the 4 directions (n = 10 trials each). The polar plot on the left shows, in black, the peak response amplitude (Ri) to each direction (θi). The thick central vector indicates the preferred angle (color coded), its length corresponds to the vector sum of the responses. The preferred direction (Dpref) was defined as: $Dpref = \arctan[\frac{\sum Risin(\theta i)}{\sum Ricos(\theta i)}]$.

(**c**) Left: 24-whisker map of direction preferences obtained for the same experiment as in **a** and **b**, for each barrel-related column. The direction preference is color coded and represented by the angle of the arrow; the length of the arrow represents the direction index: $DI = \sqrt{[\sum Risin(\theta i)]^2 + [\sum Ricos(\theta i)]^2} / \sum RI$ (DI takes values from 0 [equal responses to all directions] to 1 [complete selectivity to one direction]). Right: averaged 24-whisker map of direction preferences (n = 4 mice, white numbers indicate lower n values for some whiskers due to absent whiskers). The direction preference is color coded and represented by the angle of the arrows; the length of the arrow represents the similarity index between experiments: $SI = \frac{\sqrt{[\sum Disin(Dprefi)]^2 + [\sum Dicos(Dpref)]^2}}{\sum DI}$. The SI takes values from 0 (different Dpref between experiments) to 1 (equal Dpref). The significant anisotropic barrels (Rayleigh test; $p < 0.05$) are indicated by dotted yellow contours.

(**d**) Left: 24-whisker map illustrating the amplitudes of whisker-evoked responses averaged over the 4 directions of stimulation (n = 40 trials per whisker) for the example experiment illustrated in **a-c**. Right: averaged map from the 4 experiments (white numbers on the map indicate lower n values for some whiskers due to absent whiskers).

Figure S2

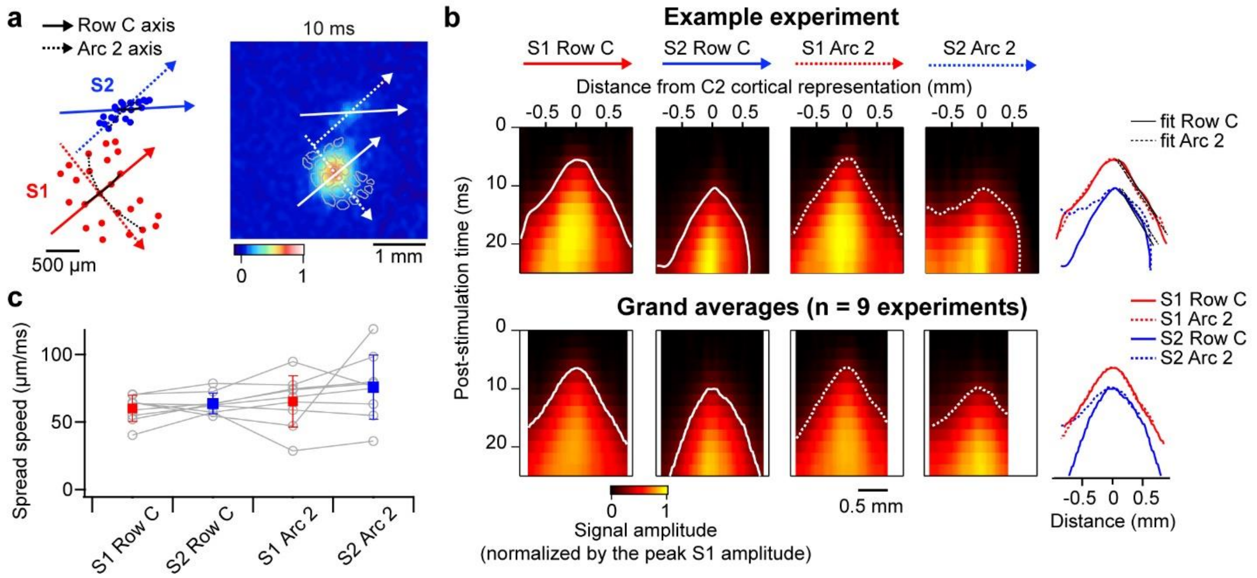

Figure S2. Spatial propagation of the C2 whisker-evoked responses is similar in S1 and S2 when quantified in absolute distance over the cortical surface.

(**a**) From the averaged S1 and S2 whisker maps shown in **Fig. 2c2**, linear fits of the spatial coordinates of the whiskers belonging to the row C and arc 2 (plain and dashed black lines, respectively) were used to define 2 axes (plain and dashed arrows, respectively) that were used to quantify the spatial propagation speed of the C2 whisker-evoked responses in absolute cortical distance (Left). For each individual experiment, these axes were translated to cross each other at the actual C2-whisker representation position in S1 and S2, as illustrated on the right, for the same example experiment as the one illustrated in **Fig. 1-4**. The row C and arc 2 axes (white plain and dashed arrows, respectively), crossing at the C2 whisker's coordinates in S1 and S2, are overlaid on an image of the averaged evoked cortical response taken 10 ms after the C2 whisker deflection (n = 10 trials, Gaussian filter 7x7 pixels), together with the histologically reconstructed S1 barrel field (grey lines).

(**b**) Upper panel: Linescan plots (distance relative to C2 on y axis and time on x axis) of the C2 whisker-evoked VSD signals quantified along the Row C and Arc 2 axes (white arrows in **a**), in S1 and S2, for the example experiment (signal normalized by the peak S1 response). A 25% threshold is shown as white plain (row C) and dashed (arc 2) lines on the linescan plots, and overlaid together on the right (in red for S1 and blue for S2). The slope of the linear fits from the starting point of the responses (shown as black lines) was used to quantify the speed of the spread. We chose to quantify the speed in only one direction (indicated by the arrows), because the proximity between S1 and S2 would otherwise impact the quantification.

(**c**) Average speed of responses spread (n = 9 experiments, mean +/- SD) quantified along the two axes in S1 (red) and S2 (blue). Values from individual experiments are shown as grey thin lines and open circles. No significant differences were observed between these values (Friedman Repeated Measures Analysis of Variance on Ranks P = 0.23).
